# Glucosylceramides trigger the shedding of alpha-synuclein laden ectosomes

**DOI:** 10.20517/evcna.2026.51

**Published:** 2026-07-20

**Authors:** Irmgard Tegeder

**Affiliations:** Institute of Clinical Pharmacology, Faculty of Medicine, Goethe University Frankfurt, Frankfurt am Main 60590, Germany.

## MAIN TEXT

Parkinson’s disease (PD) is characterized by the progressive misfolding and spreading of alpha synuclein (αSyn), which aggregates into Lewy bodies and contributes to neuronal dysfunction and loss^[1,2]^. Although traditionally viewed as a movement disorder arising from dopaminergic degeneration in the substantia nigra, PD is now recognized as a multisystem disease with peripheral and central nervous system pathology. This perspective aligns with evidence that αSyn pathology propagates between interconnected neuronal and non-neuronal cells^[3-6]^, suggesting that disease progression reflects both local vulnerability and intercellular spread.

Several routes for αSyn transfer have been identified including prion-like cell-to-cell^[7]^ and vesicle-mediated transfer and direct propagation through tunneling nanotubes (TNTs) that enable direct cytoplasmic continuity between neighboring neurons^[8]^ or neurons to glia^[9]^. TNTs contribute to the spread of αSyn fibrils and oligomers^[10]^ but were mostly recognized as a means to deliver αSyn to glia for glia-mediated degradation^[11-13]^. In contrast to TNTs, extracellular vesicles (EVs) can transport αSyn cargo over longer distances and facilitate their uptake by recipient cells^[14-16]^. These complementary pathways promote the dissemination of αSyn pathology observed in PD. The stage or region-specific relative contribution is currently unknown.

Genetic and biochemical evidence further implicates lysosomal dysfunction as a critical amplifier of αSyn toxicity. Monoallelic mutations in GBA1, the gene encoding the lysosomal enzyme glucocerebrosidase (GCase), represent the most common genetic risk factor for PD^[17-20]^. Reduced GCase activity leads to the accumulation of its substrate glucosylceramide (GlcCer), which stabilizes soluble αSyn oligomers and promotes their aggregation^[20-22]^. Conversely, increasing the αSyn burden can impair GCase trafficking and function, creating a bidirectional pathogenic loop that accelerates neurodegeneration. Emerging data suggest that GlcCer may not only influence intracellular αSyn homeostasis but also trigger an innate immune response^[23,24]^ and modulate the packaging of αSyn into EVs^[25]^ [Figure 1].

**Figure 1:**
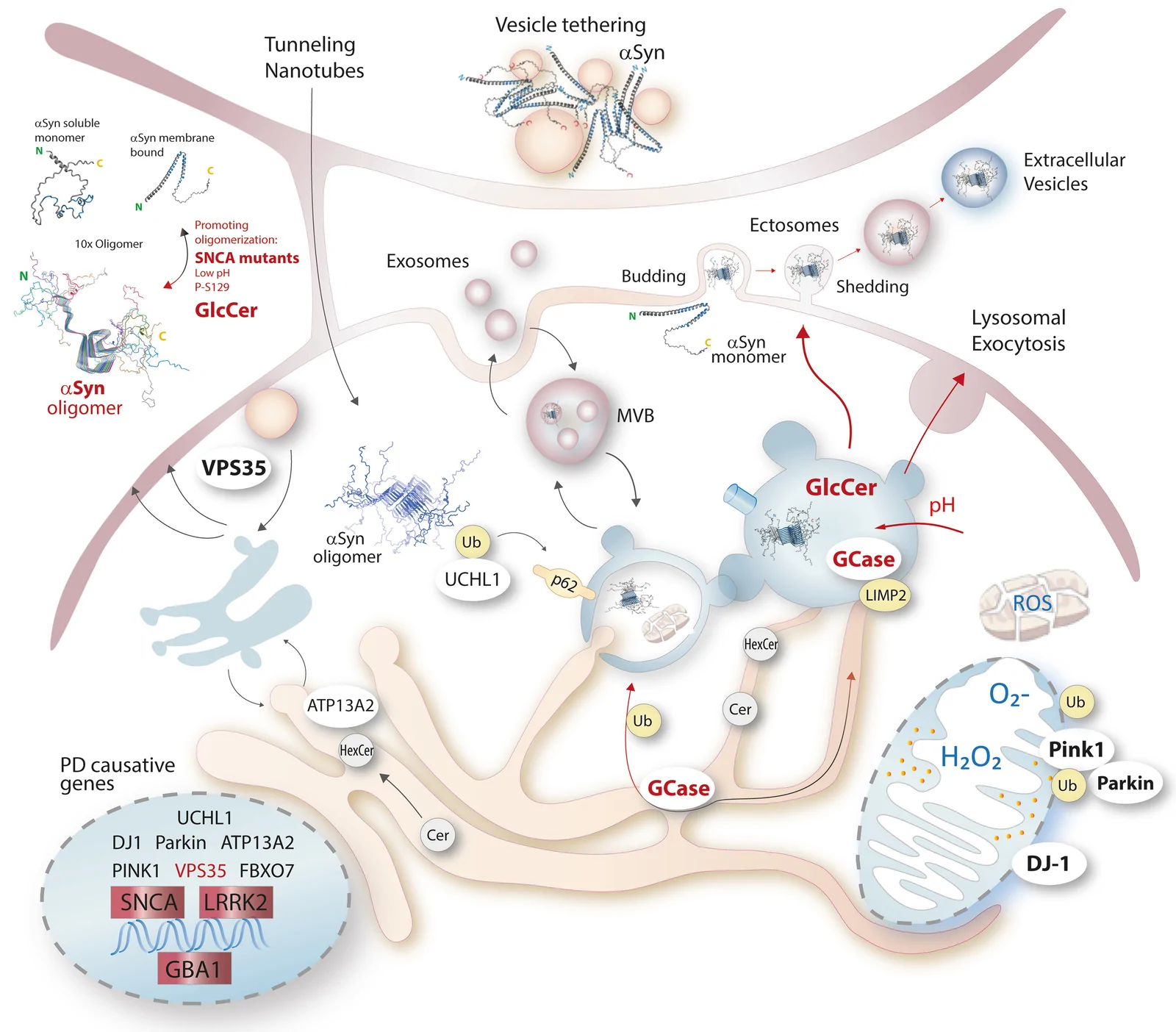
Illustration of PD genetics, the pathology of αSyn and glucosylceramides in the context of vesicle transport and release, autophagolysosomal degradation and mitochondrial damage. αSyn structure icons were obtained from PDB (micell bound monomer 1XQ8 | pdb_00001xq8^[60]^; 10x oligomer 2N0A | pdb_00002n0a)^[61]^. ATP13A2: Cation-transporting ATPase 13A2; FBXO7: F-box only protein 7; GBA1: gene encoding lysosomal glucocerebrosidase; GCase: glucocerebrosidase; LRRK2: leucine-rich repeat kinase 2; DJ-1/PARK7: protein/nucleic acid deglycase; PINK1: serine/threonine-protein kinase PINK1, mitochondrial; Parkin/Prkn: E3 ubiquitin-protein ligase parkin; p62/SQSTM1: Sequestosome-1; LIMP2/SCARB2: lysosome membrane protein 2; SNCA: alpha-synuclein; UCHL1: ubiquitin carboxyl-terminal hydrolase isozyme L1; VPS35: vacuolar protein sorting-associated protein 35; Cer: ceramides; HexCer: hexosylceramides; GlcCer: glucosylceramides; Ub: ubiquitin; MVB: multivesicular bodies; PD: Parkinson’s disease; αSyn: alpha synuclein.

A major advance comes from the recent study demonstrating that GlcCer actively drives ectosome shedding thereby promoting αSyn propagation^[25]^. The authors show that increased GlcCer, as a consequence of reduced GCase activity, induces ectosome shedding from primary neurons and that these ectosomes are loaded with αSyn and lead to the transmission of αSyn pathology to neighboring neurons. Using live cell imaging, two-photon microscopy *in vivo*, and induced pluripotent stem cells (iPSC)-derived dopaminergic neurons from patients carrying GBA1 or leucine rich repeat kinase 2 (LRRK2) mutations, the study demonstrates that GlcCer directly stimulates the outward budding and fission of large plasma membrane-derived vesicles. These ectosomes contain cytosolic markers such as tubulin, are enriched in CD81, and display the characteristic sizes expected for microvesicle-type EVs.

The authors show in primary cortical neurons that both exogenous GlcCer and pharmacological GCase inhibition with conduritol-β-epoxide increase ectosome budding without inducing cell death, showing that the ectosome release is an active, regulated process that establishes a direct link between GlcCer accumulation and vesicle-mediated αSyn spread. *In vivo*, two-photon imaging in tdTomato labeled neurons revealed ectosome formation in the intact cortex following GlcCer injection. Histology further confirmed an increased number of detached tdTomato positive vesicles, which were taken up by neighboring neurons, providing direct evidence for intercellular transfer. The study further extended these findings to human iPSC-derived dopaminergic neurons carrying GBA1 or LRRK2 mutations. All PD-associated lines exhibited reduced GCase activity and a marked increase in ectosome shedding compared with their isogenic controls. Rescue experiments, either by pharmacological enhancement of GCase activity or by overexpressing wild-type GCase, normalized ectosome release. Restoring lysosomal acidification or LRRK2 kinase inhibition also reduced ectosome formation suggesting a causal relationship between lysosomal function, GlcCer accumulation and ectosome shedding.

Together, these experiments established ectosomes rather than exosomes as a major, previously underappreciated route for αSyn transmission in PD, which was further supported experimentally showing that pharmacologic inhibition of exosome formation with GW4869 did not prevent αSyn spreading. Ectosomes are likely more efficient at transferring pathogenic αSyn than exosomes because they are larger allowing package of fibrillar αSyn and they carry plasma-membrane proteins and lipids including externally exposed phosphatidylserine that mediate rapid capture and uptake^[26-28]^ which impacts vesicle and hence, αSyn destiny^[29,30]^. Earlier studies reporting α-synuclein in EVs likely captured a mixed population of exosomes and ectosomes^[14,16,31-34]^. Given the now presented direct visualization of αSyn-laden ectosome budding^[25]^, it is plausible that ectosomes contributed substantially to the pathogenic EV cargo previously attributed to exosomes. Mixed EV populations may also explain inconsistent findings of “exosomal” αSyn as biomarker of PD^[32,35-37]^.

While the authors of^[25]^ emphasize ectosomes as a mechanism of pathogenic propagation, the same biology can also be interpreted as a neuronal clearance strategy. Neurons have limited capacity for lysosomal degradation, especially under conditions of GCase deficiency. In this context, ectosome shedding may represent a compensatory mechanism to offload toxic cytosolic cargo. The exposure of phosphatidylserine on ectosomes^[38,39]^ is a feature that flags the vesicles for phagocytosis or pinocytosis^[40,41]^, accelerating the clearance from the extracellular space and reducing the half-life of toxic cargo^[28,42-44]^. Hence, once released, glial cells are integrated into the detoxification of αSyn. This is consistent with the broader concept that EV-mediated export is part of the neuron’s stress response repertoire, analogous to lysosomal exocytosis or autophagic secretion^[45,46]^. In addition, αSyn containing EVs are detectable in peripheral body fluids^[32,37,47-49]^, suggesting that vesicular export contributes to the systemic distribution and eventually, peripheral clearance of αSyn. From this perspective, GlcCer-induced ectosome release is not solely a pathological amplifier but a dynamic, context-dependent process balancing propagation and protection.

Ambroxol, a GCase chaperone currently under clinical investigation, exemplifies this duality. It is supposed to increase the activity of mutant GCase but primarily, it triggers lysosomal exocytosis^[50-52]^. Owing to its alkaline nature it is trapped in lysosomes, increases the lysosomal pH and triggers lysosomal exocytosis^[50-52]^. Although spilling out lysosomal αSyn content may facilitate αSyn spread by receptor-mediated internalization via e.g. FAM171^[53]^ or LAG3^[54,55]^, ambroxol nonetheless produces temporary symptomatic benefits and improves cellular proteostasis^[52,56,57]^ suggesting that enhanced glial-mediated degradation outweighs the transient rise in extracellular αSyn, and that vesicle release may be protective when coupled with efficient downstream clearance.

The dual nature of ectosome biology opens intriguing therapeutic possibilities. If αSyn-laden EVs can be efficiently cleared in the periphery, then enhancing export from the brain, while simultaneously trapping or degrading vesicles outside the central nervous system (CNS), could shift the balance toward net detoxification. Several conceptual strategies emerge. Circulating αSyn EVs could be bound by engineered antibodies, nanobodies, or lipid binding polymers that selectively trap vesicles for hepatic or renal clearance or efflux through the blood-brain-barrier. Microglia and astrocytes are natural sinks for EVs. Enhancing their phagocytic capacity could accelerate the degradation of αSyn containing ectosomes before they propagate pathology. Importantly, EVs per se even without αSyn cargo or coat promote αSyn misfolding^[14,32,58]^ owing to their ceramide and curvature-rich membrane lipid domains^[59]^.

The discovery that GlcCer drives ectosome shedding provides a mechanistic bridge between lipid dysregulation, vesicle biology, and αSyn propagation. Yet, the same pathway may also serve as a neuronal survival mechanism, enabling the export of toxic cargo when intracellular degradation is compromised. Thus, the biological role of ectosomes is fundamentally dual, and their net effect depends on the balance between release and clearance.

## DECLARATIONS

### Copyright

© The Author(s) 2026.

## References

[1] McCann H, Cartwright H, Halliday GM (2016). Neuropathology of α-synuclein propagation and braak hypothesis. Mov Disord.

[2] Braak H, Del Tredici K, Rüb U, de Vos RA, Jansen Steur EN, Braak E (2003). Staging of brain pathology related to sporadic Parkinson’s disease. Neurobiol Aging.

[3] Rey NL, Steiner JA, Maroof N (2016). Widespread transneuronal propagation of α-synucleinopathy triggered in olfactory bulb mimics prodromal Parkinson’s disease. J Exp Med.

[4] Van Den Berge N, Ferreira N, Gram H (2019). Evidence for bidirectional and trans-synaptic parasympathetic and sympathetic propagation of alpha-synuclein in rats. Acta Neuropathol.

[5] Ferreira N, Gonçalves NP, Jan A (2021). Trans-synaptic spreading of alpha-synuclein pathology through sensory afferents leads to sensory nerve degeneration and neuropathic pain. Acta Neuropathol Commun.

[6] Andersen KB, Krishnamurthy A, Just MK (2025). Sympathetic and parasympathetic subtypes of body-first Lewy body disease observed in postmortem tissue from prediagnostic individuals. Nat Neurosci.

[7] Bernis ME, Babila JT, Breid S, Wüsten KA, Wüllner U, Tamgüney G (2015). Prion-like propagation of human brain-derived alpha-synuclein in transgenic mice expressing human wild-type alpha-synuclein. Acta Neuropathol Commun.

[8] Tardivel M, Bégard S, Bousset L (2016). Tunneling nanotube (TNT)-mediated neuron-to neuron transfer of pathological Tau protein assemblies. Acta Neuropathol Commun.

[9] Rostami J, Holmqvist S, Lindström V (2017). Human astrocytes transfer aggregated alpha-synuclein via tunneling nanotubes. J Neurosci.

[10] Abounit S, Bousset L, Loria F (2016). Tunneling nanotubes spread fibrillar α-synuclein by intercellular trafficking of lysosomes. EMBO J.

[11] Chakraborty R, Nonaka T, Hasegawa M, Zurzolo C (2023). Tunnelling nanotubes between neuronal and microglial cells allow bi-directional transfer of α-Synuclein and mitochondria. Cell Death Dis.

[12] Scheiblich H, Dansokho C, Mercan D (2021). Microglia jointly degrade fibrillar alpha-synuclein cargo by distribution through tunneling nanotubes. Cell.

[13] Scheiblich H, Eikens F, Wischhof L (2024). Microglia rescue neurons from aggregate-induced neuronal dysfunction and death through tunneling nanotubes. Neuron.

[14] Danzer KM, Kranich LR, Ruf WP (2012). Exosomal cell-to-cell transmission of alpha synuclein oligomers. Mol Neurodegener.

[15] Kurzawa-Akanbi M, Tammireddy S, Fabrik I (2021). Altered ceramide metabolism is a feature in the extracellular vesicle-mediated spread of alpha-synuclein in Lewy body disorders. Acta Neuropathol.

[16] Ishiguro Y, Tsunemi T, Shimada T (2024). Extracellular vesicles contain filamentous alpha-synuclein and facilitate the propagation of Parkinson’s pathology. Biochem Biophys Res Commun.

[17] Du TT, Wang L, Duan CL (2015). GBA deficiency promotes SNCA/α-synuclein accumulation through autophagic inhibition by inactivated PPP2A. Autophagy.

[18] Muñoz SS, Petersen D, Marlet FR, Kücükköse E, Galvagnion C (2021). The interplay between Glucocerebrosidase, α-synuclein and lipids in human models of Parkinson’s disease. Biophys Chem.

[19] Murphy KE, Gysbers AM, Abbott SK (2014). Reduced glucocerebrosidase is associated with increased α-synuclein in sporadic Parkinson’s disease. Brain.

[20] Mazzulli JR, Xu YH, Sun Y (2011). Gaucher disease glucocerebrosidase and α-synuclein form a bidirectional pathogenic loop in synucleinopathies. Cell.

[21] Zunke F, Moise AC, Belur NR (2018). Reversible conformational conversion of α-synuclein into toxic assemblies by glucosylceramide. Neuron.

[22] Taguchi YV, Liu J, Ruan J (2017). Glucosylsphingosine promotes α-synuclein pathology in mutant GBA-associated Parkinson’s disease. J Neurosci.

[23] Franck L, Hahnefeld L, Valek L (2025). Elevated hexosylceramides in Parkinson’s disease cause gene upregulations in neurons mimicking responses to pathogens. NPJ Parkinsons Dis.

[24] Wang R, Sun H, Cao Y (2024). Glucosylceramide accumulation in microglia triggers STING-dependent neuroinflammation and neurodegeneration in mice. Sci Signal.

[25] Jacquemyn J, Marriott B, Chang J (2026). Glucosylceramide-induced ectosomes propagate pathogenic α-synuclein in Parkinson’s disease. Nat Cell Biol.

[26] Brodeur A, Migneault F, Lanoie M (2023). Apoptotic exosome-like vesicles transfer specific and functional mRNAs to endothelial cells by phosphatidylserine-dependent macropinocytosis. Cell Death Dis.

[27] Zhang C, Yang Z, Zhou P (2021). Phosphatidylserine-exposing tumor-derived microparticles exacerbate coagulation and cancer cell transendothelial migration in triple-negative breast cancer. Theranostics.

[28] Wei X, Liu C, Wang H (2016). Surface phosphatidylserine is responsible for the internalization on microvesicles derived from hypoxia-induced human bone marrow mesenchymal stem cells into human endothelial cells. PLoS One.

[29] Abdulsahib WK, Mustafa WW, Jyothi SR (2025). Unveiling exosomes and microvesicles in Parkinson’s disease: mechanistic insights into cell death pathways and therapeutic potential. Mol Neurobiol.

[30] Cable J, Witwer KW, Coffey RJ (2023). Exosomes, microvesicles, and other extracellular vesicles-a Keystone Symposia report. Ann N Y Acad Sci.

[31] Alvarez-Erviti L, Seow Y, Schapira AH (2011). Lysosomal dysfunction increases exosome-mediated alpha-synuclein release and transmission. Neurobiol Dis.

[32] Shi M, Liu C, Cook TJ (2014). Plasma exosomal α-synuclein is likely CNS-derived and increased in Parkinson’s disease. Acta Neuropathol.

[33] Emmanouilidou E, Vekrellis K (2016). Exocytosis and spreading of normal and aberrant α-synuclein. Brain Pathol.

[34] Papadopoulos VE, Nikolopoulou G, Antoniadou I (2018). Modulation of β-glucocerebrosidase increases α-synuclein secretion and exosome release in mouse models of Parkinson’s disease. Hum Mol Genet.

[35] Jiang C, Hopfner F, Berg D (2021). Validation of α-synuclein in L1CAM-immunocaptured exosomes as a biomarker for the stratification of Parkinsonian syndromes. Mov Disord.

[36] Jiang C, Hopfner F, Katsikoudi A (2020). Serum neuronal exosomes predict and differentiate Parkinson’s disease from atypical parkinsonism. J Neurol Neurosurg Psychiatry.

[37] Niu M, Li Y, Li G (2020). A longitudinal study on α-synuclein in plasma neuronal exosomes as a biomarker for Parkinson’s disease development and progression. Eur J Neurol.

[38] Mahapatra A, Malingen SA, Rangamani P (2026). Interplay between cortical adhesion and membrane bending regulates the formation of microparticles. Soft Matter.

[39] Sun M, Xue X, Li L (2021). Ectosome biogenesis and release processes observed by using live-cell dynamic imaging in mammalian glial cells. Quant Imaging Med Surg.

[40] Bayati A, Banks E, Han C (2022). Rapid macropinocytic transfer of α-synuclein to lysosomes. Cell Rep.

[41] Park JY, Kim KS, Lee SB (2009). On the mechanism of internalization of alpha-synuclein into microglia: roles of ganglioside GM1 and lipid raft. J Neurochem.

[42] Lai RC, Lim SK (2019). Membrane lipids define small extracellular vesicle subtypes secreted by mesenchymal stromal cells. J Lipid Res.

[43] Matsumura S, Minamisawa T, Suga K (2019). Subtypes of tumour cell-derived small extracellular vesicles having differently externalized phosphatidylserine. J Extracell Vesicles.

[44] Skotland T, Llorente A, Sandvig K Lipids in extracellular vesicles: what can be learned about membrane structure and function? *Cold Spring Harb Perspect Biol* 2023;15:a041415.

[45] Xie YX, Naseri NN, Fels J (2022). Lysosomal exocytosis releases pathogenic α-synuclein species from neurons in synucleinopathy models. Nat Commun.

[46] Tsunemi T, Perez-Rosello T, Ishiguro Y (2019). Increased lysosomal exocytosis induced by lysosomal Ca^2+^ channel agonists protects human dopaminergic neurons from α-synuclein toxicity. J Neurosci.

[47] Dutta S, Hornung S, Kruayatidee A (2021). α-Synuclein in blood exosomes immunoprecipitated using neuronal and oligodendroglial markers distinguishes Parkinson’s disease from multiple system atrophy. Acta Neuropathol.

[48] Ohmichi T, Mitsuhashi M, Tatebe H, Kasai T, Ali El-Agnaf OM, Tokuda T (2019). Quantification of brain-derived extracellular vesicles in plasma as a biomarker to diagnose Parkinson’s and related diseases. Parkinsonism Relat Disord.

[49] Sepúlveda D, Cisternas-Olmedo M, Arcos J, Nassif M, Vidal RL (2022). Contribution of autophagy-lysosomal pathway in the exosomal secretion of alpha-synuclein and its impact in the progression of Parkinson’s disease. Front Mol Neurosci.

[50] Magalhaes J, Gegg ME, Migdalska-Richards A, Schapira AH (2018). Effects of ambroxol on the autophagy-lysosome pathway and mitochondria in primary cortical neurons. Sci Rep.

[51] Fois G, Hobi N, Felder E (2015). A new role for an old drug: ambroxol triggers lysosomal exocytosis via pH-dependent Ca^2+^ release from acidic Ca^2+^ stores. Cell Calcium.

[52] Franck L, Valek L, Hahnefeld L (2026). Modest improvement of metabolic and behavioral deficits with long-term ambroxol treatment in a Pink1^-/-^SNCA^A53T^ double mutant mouse model of Parkinson’s disease. Acta Pharmacol Sin.

[53] Wu KM, Xu QH, Liu YQ (2025). Neuronal FAM171A2 mediates α-synuclein fibril uptake and drives Parkinson’s disease. Science.

[54] Mao X, Ou MT, Karuppagounder SS (2016). Pathological α-synuclein transmission initiated by binding lymphocyte-activation gene 3. Science.

[55] Zhang Q, Duan Q, Gao Y (2023). Cerebral microvascular injury induced by lag3-dependent α-synuclein fibril endocytosis exacerbates cognitive impairment in a mouse model of α-synucleinopathies. Adv Sci.

[56] Ambrosi G, Ghezzi C, Zangaglia R, Levandis G, Pacchetti C, Blandini F (2015). Ambroxol-induced rescue of defective glucocerebrosidase is associated with increased LIMP-2 and saposin C levels in GBA1 mutant Parkinson’s disease cells. Neurobiol Dis.

[57] Colucci F, Avenali M, De Micco R (2023). Ambroxol as a disease-modifying treatment to reduce the risk of cognitive impairment in GBA-associated Parkinson’s disease: a multicentre, randomised, double-blind, placebo-controlled, phase II trial. The AMBITIOUS study protocol. BMJ Neurol Open.

[58] Stuendl A, Kunadt M, Kruse N (2016). Induction of α-synuclein aggregate formation by CSF exosomes from patients with Parkinson’s disease and dementia with Lewy bodies. Brain.

[59] Ugalde CL, Gordon SE, Shambrook M (2020). An intact membrane is essential for small extracellular vesicle-induced modulation of α-synuclein fibrillization. J Extracell Vesicles.

[60] Ulmer TS, Bax A, Cole NB, Nussbaum RL (2005). Structure and dynamics of micelle-bound human alpha-synuclein. J Biol Chem.

[61] Tuttle MD, Comellas G, Nieuwkoop AJ (2016). Solid-state NMR structure of a pathogenic fibril of full-length human α-synuclein. Nat Struct Mol Biol.

